# Relationship between transformational leadership and positive youth development in Japanese sports

**DOI:** 10.3389/fspor.2025.1569274

**Published:** 2025-04-25

**Authors:** Saori Nakayama, Makoto Izawa

**Affiliations:** ^1^ Institute of Health and Sport Sciences, University of Tsukuba, Ibaraki, Japan; ^2^ School of Physical Education, Health and Sport Sciences, University of Tsukuba, Ibaraki, Japan

**Keywords:** sports coaching, coach-athlete relationships, youth sports, performance expectations, Japanese sports culture

## Abstract

**Introduction:**

This study examined the relationship between transformational leadership (TFL) behaviors of coaches and positive youth development (PYD) outcomes in Japanese youth sports settings. Research has shown associations between TFL and various athlete outcomes in Western sports contexts. However, limited attention has been paid to how cultural factors shape these relationships in East Asian settings, particularly within Japanese sports culture that emphasizes hierarchical relationships and collectivist values.

**Methods:**

The study included 112 first-year undergraduate students from a sports science program at a Japanese university. Athletes' perceptions of their coaches' TFL behaviors and PYD outcomes were assessed using the Differentiated Transformational Leadership Inventory for Youth Sport (DTLI-YS) and the Youth Experience Survey for Sport (YES-S).

**Results:**

Correlation analyses revealed a particularly strong association between high performance expectations and initiative (*r* = .53, *p* < .01), notable when compared to American research where correlations between PYD outcomes and TFL dimensions were typically weaker (*r* = .11 – .43). Regression analyses further showed that high performance expectations were significantly associated with both goal setting (*β* = .29, *p* < .05) and initiative (*β* = .39, *p* < .01). Analysis by competition level revealed significant differences in initiative (*F* = 4.07, *p* < .01, *η*² = .10) and total YES-S scores (*F* = 2.75, *p* < .05, *η*² = .07).

**Discussion:**

These findings contribute to understanding how cultural context shapes the relationship between coaching leadership and youth development in sports. While the prominence of high performance expectations reflects Japanese sports culture's emphasis on discipline and collective achievement, results suggest the importance of incorporating a more balanced leadership approach that includes elements beyond high performance expectations to foster comprehensive athlete development.

## 1 Introduction

Over the past decade, the role of sports activities in youth development has received increasing scholarly attention worldwide. Within this context, Positive Youth Development through Sport (PYD) has emerged as a theoretical framework that emphasizes the development of core competencies essential for future social contribution, encompassing personal and social skills, cognitive abilities, and goal-setting capacities (1). While the PYD framework provides a valuable approach for understanding developmental outcomes in sports, critical perspectives have also emphasized the importance of examining the specific conditions and cultural contexts under which sports participation contributes to positive development, rather than assuming universal developmental benefits (2). Empirical evidence indicates that sports participation contributes to positive behavioral, psychological, and social health outcomes in individuals aged 5–25 years (3).

Within this developmental framework, Transformational Leadership (TFL) has emerged as a particularly salient construct in youth sport research (4). TFL is a leadership approach that emphasizes inspiring followers to exceed expected performance by fostering motivation, commitment, and engagement through idealized influence, inspirational motivation, intellectual stimulation, and individualized consideration (5). When applied to sport contexts, TFL theory has been refined to include six transformational components—individualized consideration, inspirational motivation, intellectual stimulation, fostering acceptance of group goals and teamwork, high performance expectations, appropriate role model—complemented by contingent reward as a transactional element (6). Within this framework, transformational coaches inspire athletes to transcend self-interest for team goals, develop their full potential, and achieve outcomes beyond expectations. Empirical studies in Western contexts have demonstrated that these leadership dimensions are associated with multiple positive outcomes, including enhanced intrinsic motivation (7), increased team cohesion (6), elevated athlete well-being (8), and enhanced competitive performance (9).

Evidence supporting the efficacy of TFL in youth sports continues to accumulate across diverse contexts. A study of Australian youth soccer revealed that athletes coached under TFL principles reported enhanced developmental experiences (10). Subsequently, investigations of American youth basketball demonstrated positive associations between specific TFL elements—notably individualized consideration, intellectual stimulation, and exemplary conduct—and developmental outcomes as measured by the Youth Experience Survey for Sport (YES-S) (11). Additional support comes from research in Norwegian elite youth soccer, where TFL behaviors were positively associated with multiple adaptive outcomes, including task and social cohesion, mastery-oriented motivational climate, self-regulated learning processes, and athlete satisfaction, while showing inverse relationships with performance-focused motivational orientations (12).

Despite cultural differences and unique coaching environments, limited research has examined the relationship between coaches’ leadership styles and PYD in Japanese sports settings. In particular, the impact of distinctly Japanese elements such as emphasis on hierarchical relationships and collectivist values (13) on TFL effectiveness remains unexplored.

It is important to note that Japanese sports culture embodies unique elements that may influence the effectiveness of coaching leadership styles. One such concept is “konjo” (spirit or grit), which represents a distinctive psychological quality in Japanese culture that encompasses mental fortitude and indomitable fighting spirit developed through sports participation (14). While occasionally translated into English as “guts” to convey determination and courage, this translation fails to capture the full cultural significance of the original Japanese term. In Japanese sports contexts, the concept of konjo has traditionally shaped hierarchical coach-athlete relationships, potentially influencing how TFL components function within this environment (15). Understanding these cultural nuances is essential for contextualizing the application of Western leadership theories in Japanese sports settings.

This study aims to elucidate the relationship between coaches’ leadership styles and athletes’ positive development in the Japanese sports environment. Specifically, we focus on how the seven components of TFL function within the Japanese cultural context, empirically examining their relationships with athlete development.

## 2 Method

### 2.1 Participants

Participants were 112 first-year undergraduate students (71 males, 41 females) enrolled in the Department of Sports Science at a large public university in the Kanto area of Japan. The mean age of participants was 18.30 years (SD = 0.99). All participants were Japanese. The department is characterized by high-performing clubs in domestic collegiate competitions and students who compete internationally at various levels, including age-group and senior national teams. Following Côté and Gilbert’s (16) coaching context typology, these students likely received coaching from performance-focused coaches during high school. This suggests both deep athletic commitment and coaches’ emphasis on performance variables.

Participants represented a diverse range of sports, including track and field (*n* = 25), kendo (*n* = 6), swimming (*n* = 5), tennis (*n* = 4), gymnastics (*n* = 4), badminton (*n* = 2), diving (*n* = 1), aerobics (*n* = 1), dance (*n* = 7), baseball (*n* = 21), soccer (*n* = 10), volleyball (*n* = 9), handball (*n* = 8), basketball (*n* = 7), and rugby (*n* = 2). This distribution encompasses both individual and team sports, as well as sports with varying degrees of traditional Japanese influence vs. Western sporting traditions, allowing for a comprehensive analysis of leadership relationships across different sporting contexts.

### 2.2 Procedure

The survey was administered at the end of a regular class session in June 2024. Students were approached during their “Freshman seminar” class, with the professor's permission. All 195 students present were invited to participate, with 112 completing the survey (62.3% response rate). Participants took an average of 7.5 min (SD = 2.3) to complete the survey. Prior to data collection, participants were informed about the study's purpose and significance, voluntary nature of participation, right to withdraw, anonymity, and data protection procedures. Data were collected using Google Forms, with form submission constituting implied consent.

### 2.3 Measures

#### 2.3.1 Transformational leadership

Athletes’ perceptions of their coach's TFL behaviors were assessed using the Differentiated Transformational Leadership Inventory for Youth Sport (DTLI-YS; 17). We employed the Japanese version of DTLI-YS (18), which comprises six TFL factors (individualized consideration, inspirational motivation, intellectual stimulation, fostering acceptance of group goals and teamwork, high performance expectations, appropriate role model) and one transactional leadership factor (contingent reward).

The participants responded to 28 items on a 5-point Likert scale ranging from 1 (not at all) to 5 (all the time). Each item began with “My coach.” Example items included “My coach recognises that different athletes have different needs” (individual consideration subscale) and “My coach gets me to rethink the way that I do things” (intellectual stimulation subscale). Internal consistency reliability was satisfactory for all subscales, with Cronbach's *α* coefficients exceeding .74, above the recommended threshold of .70 (19).

#### 2.3.2 Positive youth development outcomes

Positive youth development (PYD) outcomes based on sports participation experiences were measured using Youth Experience Survey for Sport (YES-S; 20). Participants responded to 27 statements reflecting on their high school sports experiences across four subscales (personal and social skills, cognitive skills, goal setting, initiative), rated on a 4-point Likert scale ranging from 1 (not at all) to 4 (definitely). Example items included “I became better at taking feedback” (personal/social skills) and “Learned to find ways to reach my goals” (initiative). Ten negative experience items from the original YES-S were excluded from the survey to reduce response burden. These items constituted the “Negative Experiences” subscale and included statements such as “Adult leaders in this activity were controlling and manipulative” and “Youth in this activity got me into drinking alcohol or using drugs.” This decision was made because (1) our primary focus was on positive developmental outcomes, (2) previous research has demonstrated that the four positive subscales function independently from the negative subscale (20), and (3) to maintain a manageable survey length for participants simultaneously responding to multiple measures.

Internal consistency reliability analysis revealed Cronbach's *α* coefficients of 0.84 for personal and social skills, 0.76 for cognitive skills, 0.72 for goal setting, and 0.67 for initiative (Table 1). Although this value in initiative falls slightly below the conventional 0.70 threshold, it was considered acceptable given the limited number of items in the subscale. As noted by Shimizu and Shojima (21), Cronbach's α coefficients tend to decrease with fewer items. Since the initiative subscale contained only three items, the α coefficient below 0.7 can be attributed to this limited number of items rather than poor internal consistency (22).

**Table 1:** Internal consistency reliability analysis of each scale.

Scales and subscales	Cronbach's *α*
YES-S	
Personal and social skills	0.84
Cognitive skills	0.76
Goal setting	0.72
Initiative	0.67
DTLI-YS	
Individualized consideration	0.81
Inspirational motivation	0.74
Intellectual stimulation	0.79
Fostering acceptance of group goals and teamwork	0.78
High performance expectations	0.80
Appropriate role model	0.81
Contingent reward	0.88

Note: YES-S, youth experience survey for sport; DTLI-YS, differentiated transformational leadership inventory for youth sport.

#### 2.3.3 Attributes

Participants reported their highest level of sports achievement during high school, categorized as district/prefectural level, regional level, national level, and national top 8 placements. Demographic data included age and gender (options: male, female, prefer not to answer).

### 2.4 Statistical analysis

Statistical analyses were conducted using IBM SPSS Statistics version 29.0.2.0. Relationships between variables were examined using Pearson correlation coefficients, with coefficients ≥ 0.5 considered meaningful. Statistical significance was set at *p* < .01 and *p* < .05. Gender differences were assessed using independent *t*-tests, with effect sizes calculated using Cohen's *d* (0.2 = small, 0.5 = medium, 0.8 = large) (23). Differences across competition levels were analyzed using one-way analysis of variance (ANOVA), followed by Tukey's HSD tests for significant findings. Effect sizes were calculated using eta squared (*η*^2^), with values of 0.01, 0.06, and 0.14 representing small, medium, and large effects, respectively (23). The influence of DTLI-YS subscales on YES-S outcomes was investigated using multiple regression analysis.

To evaluate the adequacy of our sample size, we conducted *post-hoc* power analyses using G*Power 3.1 (24). For multiple regression analyses with seven predictors (DTLI-YS dimensions), given the observed effect sizes (*f*^2^ = 0.176 for personal and social skills, *f*^2^ = 0.190 for cognitive skills, *f*^2^ = 0.250 for goal setting, and *f*^2^ = 0.408 for initiative) and an *α* level of .05, the statistical power ranged from 0.90 to 0.99. For ANOVA analyses examining competition level differences (four groups), with observed effect sizes of *η*^2^ = 0.10 for initiative and *η*^2^ = 0.07 for total YES-S scores, the statistical power was 0.84 and 0.66, respectively. For the independent samples *t*-test examining gender differences in cognitive skills (*n*₁ = 71, *n*₂ = 41), with an effect size of *d* = 0.62, the statistical power was 0.93. These values exceed the recommended power threshold of .80 (23) for most analyses, suggesting that our sample size was generally sufficient, although a larger sample would be beneficial for detecting differences in total YES-S scores across competition levels.

## 3 Results

### 3.1 Descriptive statistics

Means and standard deviations for all variables are presented in Table 2. Among YES-S subscales, initiative demonstrated the highest mean score, while cognitive skills showed the lowest. For DTLI-YS dimensions, individualized consideration exhibited the highest mean score, with appropriate role model showing the lowest.

**Table 2:** Means and standard deviations for scales.

Variable	Mean (SD)
YES-S	2.88 (0.46)
Personal and social skills	2.84 (0.48)
Cognitive skills	2.51 (0.63)
Goal setting	2.98 (0.61)
Initiative	3.18 (0.63)
DTLI-YS	4.06 (0.68)
Individualized consideration	4.25 (0.79)
Inspirational motivation	4.01 (0.75)
Intellectual stimulation	3.93 (0.81)
Fostering acceptance of group goals and teamwork	4.08 (0.88)
High performance expectations	4.20 (0.79)
Appropriate role model	3.82 (0.93)
Contingent reward	4.15 (0.85)

Note: YES-S, youth experience survey for sport; DTLI-YS, differentiated transformational leadership inventory for youth sport.

### 3.2 Correlation analysis

Bivariate correlations between scales and subscales are shown in Table 3. The total YES-S score showed a significant positive correlation with the total DTLI-YS score (*r* = .49, *p* < .01). YES-S subscales showed significant moderate to strong positive intercorrelations (*r* = .56–.73, *p* < .01). Similarly, DTLI-YS subscales demonstrated significant moderate to strong positive intercorrelations (*r* = .39–.76, *p* < .01), with particularly strong associations between individualized consideration and contingent reward (*r* = .76, *p* < .01).

**Table 3:** Correlation coefficients between variables.

Variable	1	1a	1b	1c	1d	2	2a	2b	2c	2d	2e	2f	2g
1. YES-S	-												
1a. Personal and social skills	**0** **.** **92** **	-											
1b. Cognitive Skills	**0** **.** **88** **	**0** **.** **73** **	-										
1c. Goal Setting	**0** **.** **84** **	**0** **.** **67** **	**0** **.** **72** **	-									
1d. Initiative	**0** **.** **71** **	**0** **.** **56** **	**0** **.** **57** **	**0** **.** **64** **	-								
2. DTLY-YS	0.49**	0.39**	0.45**	0.48**	**0** **.** **50** **	-							
2a. Individualized consideration	0.39**	0.29**	0.33**	0.43**	0.36**	**0** **.** **86** **	-						
2b. Inspirational motivation	0.35**	0.25**	0.36**	0.36**	0.42**	**0** **.** **82** **	**0** **.** **68** **	-					
2c. Intellectual stimulation	0.46**	0.38**	0.41**	0.45**	0.40**	**0** **.** **85** **	**0** **.** **74** **	**0** **.** **60** **	-				
2d. Fostering acceptance of group goals and teamwork	0.41**	0.35**	0.37**	0.38**	0.46**	**0** **.** **84** **	**0** **.** **63** **	**0** **.** **66** **	**0** **.** **65** **	-			
2e. High performance expectations	0.45**	0.38**	0.33**	0.44**	**0** **.** **53** **	**0** **.** **69** **	0.47**	0.43**	**0** **.** **52** **	**0** **.** **67** **	-		
2f. Appropriate role model	0.45**	0.38**	0.42**	0.36**	0.39**	**0** **.** **85** **	**0** **.** **63** **	**0** **.** **63** **	**0** **.** **72** **	**0** **.** **66** **	**0** **.** **54** **	-	
2g. Contingent reward	0.33**	0.23*	0.32**	0.32**	0.31**	**0** **.** **84** **	**0** **.** **76** **	**0** **.** **72** **	**0** **.** **64** **	**0** **.** **63** **	0.39**	**0** **.** **65** **	-

Note: Bold numbers indicate *r* > 0.5. YES-S, youth experience survey for sport; DTLI-YS, differentiated transformational leadership inventory for youth sport.

* *p* < .05.

** *p* < .01.

Analyses of cross-scale relationships revealed significant weak to moderate positive correlations between DTLI-YS subscales and YES-S subscales (*r* = .23–.53, *p* < .05). Of note, high performance expectations demonstrated a relatively strong association with initiative (*r* = .53, *p* < .01). The total DTLI-YS score showed a moderate positive correlation with initiative (*r* = .50, *p* < .01).

### 3.3 Gender differences

Gender comparisons for all variables are presented in Table 4. The sample consisted of 71 males (63.4%) and 41 females (36.6%). A significant gender difference emerged only for cognitive skills, with male participants (*M* = 2.61, SD = 0.63) scoring higher than female participants (*M* = 2.35, SD = 0.60; *t* = 2.13, *p* = .04, *d* = 0.62), representing a medium effect size.

**Table 4:** Comparison of scale scores by gender.

Variable	Male mean (SD)	Female mean (SD)	*t*-value	*p*-value	Cohen's *d*
	(*n* = 71)	(*n* = 41)			
YES-S	2.92 (0.47)	2.82 (0.43)	1.04	0.30	0.47
Personal and social skills	2.83 (0.51)	2.86 (0.43)	−0.35	0.73	0.48
Cognitive skills	2.61 (0.63)	2.35 (0.60)	2.13	0.04*	0.62
Goal setting	3.05 (0.61)	2.86 (0.60)	1.59	0.11	0.61
Initiative	3.25 (0.60)	3.06 (0.65)	1.61	0.11	0.62
DTLI-YS	4.12 (0.68)	3.94 (0.66)	1.40	0.17	0.67
Individualized consideration	4.30 (0.79)	4.18 (0.79)	0.79	0.43	0.79
Inspirational motivation	4.09 (0.72)	3.87 (0.80)	1.48	0.14	0.75
Intellectual stimulation	4.01 (0.85)	3.80 (0.73)	1.31	0.19	0.81
Fostering acceptance of group goals and teamwork	4.16 (0.82)	3.93 (0.96)	1.39	0.17	0.87
High performance expectations	4.24 (0.74)	4.13 (0.87)	0.72	0.47	0.79
Appropriate role model	3.92 (0.90)	3.65 (0.97)	1.48	0.14	0.93
Contingent reward	4.21 (0.76)	4.02 (0.98)	1.07	0.29	0.85

Note: YES-S, youth experience survey for sport; DTLI-YS, differentiated transformational leadership inventory for youth sport. Cohen's *d*: 0.2 = small, 0.5 = medium, 0.8 = large.

* *p* < .05.

### 3.4 Competition level differences

The sample included participants with varying levels of competitive experience during high school. Of the 112 participants, 24 (21.4%) competed at the district/prefecture level, 27 (24.1%) at the regional level, 38 (33.9%) at the national level, and 23 (20.5%) achieved national top 8 placements. Comparisons across competition levels (district/prefecture, regional, national, national top 8) are presented in Table 5. One-way ANOVA revealed significant main effects for initiative (*F* = 4.07, *p* < .01, *η*^2^ = .10, medium effect) and total YES-S score (*F* = 2.75, *p* < .05, *η*^2^ = .07, medium effect). *Post-hoc* Tukey's tests indicated that participants who competed at the national top 8 level reported significantly higher initiative scores than those competing at the district/prefecture level. Although other YES-S subscales and TFL subscales did not demonstrate statistically significant differences, mean scores tended to increase with ascending competition levels.

**Table 5:** Comparison of scale scores by highest high school achievement level.

Variable	District/Prefecture^a^ (SD)	Regional^b^ (SD)	National^c^ (SD)	National Top 8^d^ (SD)	*F*	*p*	*η* ^2^	Tukey's HSD *post-hoc* test
	(*n* = 34)	(*n* = 18)	(*n* = 22)	(*n* = 36)				
YES-S	2.79 (0.41)	2.77 (0.39)	2.91 (0.44)	3.04 (0.47)	2.75	0.05*	0.07	
Personal and social skills	2.77 (0.43)	2.75 (0.38)	2.84 (0.47)	2.89 (0.52)	1.62	0.19	0.04	
Cognitive skills	2.42 (0.57)	2.39 (0.62)	2.52 (0.58)	2.72 (0.66)	1.91	0.13	0.05	
Goal setting	2.85 (0.54)	2.90 (0.58)	3.07 (0.53)	3.16 (0.65)	1.97	0.12	0.05	
Initiative	2.98 (0.59)	3.07 (0.52)	3.36 (0.65)	3.40 (0.52)	4.07	0.01**	0.10	d > a
DTLI-YS	3.92 (0.66)	3.97 (0.72)	4.02 (0.71)	4.28 (0.61)	1.90	0.13	0.05	
Individualized consideration	4.22 (0.75)	4.06 (1.10)	4.31 (0.74)	4.38 (0.66)	0.71	0.55	0.02	
Inspirational motivation	3.81 (0.80)	3.85 (0.85)	4.11 (0.58)	4.24 (0.71)	2.47	0.07	0.07	
Intellectual stimulation	3.86 (0.84)	3.82 (0.77)	3.91 (0.95)	4.11 (0.72)	0.78	0.51	0.02	
Fostering acceptance of group goals and teamwork	3.82 (0.94)	4.15 (0.78)	4.12 (0.92)	4.35 (0.69)	2.37	0.08	0.06	
High performance expectations	4.13 (0.69)	4.18 (0.72)	4.09 (0.89)	4.47 (0.66)	1.78	0.16	0.05	
Appropriate role model	3.68 (0.93)	3.72 (0.81)	3.59 (1.04)	4.14 (0.85)	2.27	0.09	0.06	
Contingent reward	4.01 (0.89)	4.08 (0.94)	4.09 (0.78)	4.33 (0.81)	0.90	0.44	0.03	

Note: The exact *p*-value for YES-S total score was .046. *η*^2^ = 0.01 Small, 0.06 medium, 0.14 large. YES-S, youth experience survey for sport; DTLI-YS, differentiated transformational leadership inventory for youth sport. Different superscript letters indicate significant differences between groups (*p* < .05) using Tukey's HSD *post-hoc* test.

* *p* < .05.

** *p* < .01.

### 3.5 Multiple regression analysis

Results of multiple regression analyses examining the relationship between TFL components and PYD outcomes are presented in Table 6. All regression models demonstrated significant predictive power (Adjusted *R*^2^ ranging from .15 to .29, all *p* < .01), with initiative showing the highest proportion of explained variance (Adjusted *R*^2^ = .29). High performance expectations emerged as a significant predictor of both goal setting (*β* = .29, *p* < .05) and initiative (*β* = .39, *p* < .01). However, no TFL components significantly predicted personal and social skills or cognitive skills.

**Table 6:** Multiple regression analysis.

DTLI-YS subscales	Personal and social skills		Cognitive skills		Goal setting		Initiative	
	*β*	*B*	*β*	*B*	*β*	*B*	*β*	*B*
Individualized consideration	−0.01	−0.01	−0.05	−0.04	0.19	0.15	−0.01	−0.01
Inspirational motivation	−0.04	−0.02	0.13	0.11	0.10	0.08	0.26	0.21
Intellectual stimulation	0.21	0.12	0.18	0.14	0.22	0.17	0.08	0.07
Fostering acceptance of group goals and teamwork	0.06	0.03	0.04	0.03	−0.07	−0.05	0.03	0.02
High performance expectations	0.20	0.12	0.10	0.08	0.29*	0.22	0.39**	0.31
Appropriate role model	0.16	0.08	0.17	0.12	−0.04	−0.02	0.00	0.00
Contingent reward	−0.09	−0.05	−0.02	−0.01	−0.09	−0.06	−0.09	−0.07
Adjusted *R*²		0.15**		0.16**		0.23**		0.29**
*F* value		3.87		4.04		5.72		7.34

Note: *β* Represents standardized regression coefficients, *B* represents unstandardized regression coefficients. Adjusted *R*^2^ values are reported for regression models.

* *p* < .05.

** *p* < .01.

## 4 Discussion

### 4.1 Cultural context and leadership impact

Regression analyses (Table 6) demonstrate that among TFL components, high performance expectations emerged as a significant predictor of both goal setting (*β* = .29, *p* < .05) and initiative (*β* = .39, *p* < .01). Our correlation analyses revealed a moderate positive correlation between the total DTLI-YS score and the total YES-S score (*r* = .49, *p* < .01), indicating a substantial relationship between TFL behaviors as a whole and PYD outcomes overall. This finding aligns with the growing body of research supporting the effectiveness of TFL approaches in fostering positive developmental experiences in youth sports contexts.

Furthermore, our correlation analyses revealed a particularly strong association between high performance expectations and initiative (*r* = .53, *p* < .01; Table 3). This finding is notable when compared to American research (11), where correlations between PYD outcomes and TFL dimensions were all below *r* = .5, showing only weak to moderate associations (*r* = .11–.43). In contrast, our study identified high performance expectations as having a distinctively strong relationship with PYD outcomes, particularly initiative, in the Japanese sporting context.

One theoretical framework that may help explain these findings is the Pygmalion effect. According to Rejeski et al. (25), coaches’ expectations can significantly influence athlete behavior and performance outcomes. Their landmark study found that high-expectancy athletes received more reinforcement and feedback, while low-expectancy athletes experienced different interaction patterns, including more general technical instruction. When coaches communicate high expectations, they often provide more attention, feedback, and encouragement to athletes they believe will succeed, creating a self-fulfilling prophecy that enhances athletes’ proactive engagement. In Japanese culture, where respect for authority is highly valued, the Pygmalion effect may be even stronger. Japanese athletes may be more likely to accept and internalize their coaches’ high expectations, leading to greater initiative and effort in their sporting activities.

These findings align with traditional Japanese sports coaching culture, which has historically emphasized “konjo” (spirit or grit)—a concept that values perseverance through hardship and meeting strict expectations (15). This cultural emphasis on discipline aligns with our finding that high performance expectations significantly predict initiative and goal setting. In contrast to Western coaching philosophies that often emphasize athlete autonomy and individualized approaches (4), Japanese coaching traditionally places greater emphasis on hierarchical relationships and collective achievement (13).

However, our findings indicate that performance expectations alone may not be sufficient for comprehensive athlete development. The absence of significant relationships between other leadership components (e.g., intellectual stimulation) and personal and social or cognitive skills development (Table 6) suggests the need to integrate approaches that foster athletes’ autonomous thinking and decision-making capabilities alongside traditional expectation-based coaching methods. Natsubara et al. (26) highlighted the importance of considering cultural contexts when examining TFL in sports, and our findings contribute to this growing body of research by identifying how specific leadership dimensions function differently within Japanese sporting environments.

### 4.2 Coach-athlete relationships in Japanese context

Beyond the broader cultural context discussed above, the characteristics of coach-athlete relationships also serves as a crucial element shaping the effectiveness of TFL in Japanese sports environments. Our analyses revealed a striking contrast between the influence of high performance expectations and appropriate role model on PYD outcomes. While high performance expectations demonstrated a relatively stronger association with initiative (*r* = .53, *p* < .01) and significantly predicted both initiative (*β* = .39, *p* < .01) and goal setting (*β* = .29, *p* < .05), appropriate role model showed only modest relationships with PYD outcomes, with correlation coefficients not exceeding *r* = 0.42 (Table 3). Notably, appropriate role model failed to emerge as a significant predictor for any PYD outcome in the regression analysis (Table 6).

This finding suggests a nuanced dynamic in the coach-athlete relationship where athletes respond positively to coaches’ high expectations while showing less robust identification with them as role models. This pattern aligns with Japanese research on coach-athlete relationships. Yamaguchi et al. (27) found that Japanese high school judo athletes reported lower levels of closeness, commitment, and complementarity with their coaches compared to athletes from seven other countries. Similarly, Okada et al. (28, 29) found that Japanese high school judo athletes’ trust in their coaches was considerably lower than that of Australian university athletes. Both studies suggest that Japanese athletes often lack strong trust in or closeness to their coaches, despite being responsive to their expectations. This contrasts with Western coaching models that emphasize mutual trust and role modeling. The effectiveness of high performance expectations within this context suggests that Japanese athletes may respond to hierarchical expectations as a cultural norm rather than through personal identification with their coaches. These findings suggest that in Japanese sporting contexts, performance expectations may function effectively even without close personal connections, highlighting the need for culturally-sensitive leadership approaches in sports.

### 4.3 Gender differences in development outcomes

Male participants scored significantly higher on cognitive skills (*M* = 2.61, SD = 0.63) than female participants (*M* = 2.35, SD = 0.60; *t* = 2.13, *p* = .04, *d* = 0.62; Table 4). This gender difference in cognitive skills is consistent with D. O'Connor et al. (30), who reported higher scores for boys (*M* = 2.52, SD = 0.80) compared to girls (*M* = 2.23, SD = 0.73; *p* = .01). However, these results require careful interpretation given the measurement scale’s characteristics. As noted by Cronin and Allen (31), YES-S cognitive skill items may not fully capture sport-specific cognitive competencies, such as tactical thinking, situational judgment, and opponent analysis. Such measurement limitations could particularly influence female athletes’ self-evaluation. No significant gender differences were found in other YES-S subscales (personal and social skills, goal setting, initiative) or any TFL subscales. These findings suggest that many developmental outcomes of sports participation may be similarly experienced across genders, supporting previous research (32) indicating gender-independent developmental benefits of sport participation. This absence of significant gender differences in most developmental outcomes challenges persistent stereotypes about gendered abilities in sports settings and suggests that when provided with appropriate leadership and opportunities, male and female athletes may develop similar competencies through sports participation.

### 4.4 Competition level effects

Competition levels showed significant effects on initiative (*F* = 4.07, *p* < .01, *η*^2^ = .10) and overall YES-S scores (*F* = 2.75, *p* < .05, *η*^2^ = .07; Table 5). *Post-hoc* analyses indicated that national top 8 performers scored significantly higher on initiative than district/prefecture level athletes. These findings suggest that success at higher competitive levels may be associated with developing self-directed engagement in training and competition.

The relationship between initiative and competitive level may be bidirectional: higher initiative might contribute to competitive success, while high-level competitive experiences might foster initiative through exposure to complex tactical demands and advanced technical challenges requiring independent problem-solving skills.

No significant differences emerged in TFL subscales across competition levels (Table 5), suggesting that coaches’ leadership styles do not substantially differ across competitive levels. However, small to medium effect sizes were observed for inspirational motivation (*η*^2^ = .07) and fostering acceptance of group goals and teamwork (*η*^2^ = .06), suggesting potential relationships that might reach statistical significance with larger samples.

### 4.5 Research limitations and future directions

This study has several limitations that warrant consideration and suggest directions for future research. The cross-sectional design constrains our ability to make causal inferences, highlighting the need for longitudinal studies to establish temporal relationships between leadership styles and developmental outcomes. Additionally, as the sample consisted exclusively of university students, validation across different age groups and competitive levels is necessary to enhance generalizability. The unique aspects of the Japanese coaching environment, particularly within the school sports system, require further investigation to understand their specific influences on the observed relationships.

*Post-hoc* power analyses revealed that while our sample size (*n* = 112) was sufficient for detecting effects in multiple regression analyses (power = 0.90–0.99) and for initiative in ANOVA (power = 0.84), it was somewhat underpowered for detecting differences in total YES-S scores across competition levels (power = 0.66). This suggests that future research examining comprehensive developmental outcomes across competition levels should employ larger samples to increase statistical power.

Despite including participants from various sports (track and field, kendo, swimming, baseball, soccer, volleyball, etc.), the relatively small subsamples for each sport (ranging from *n* = 1–25) prevented meaningful sport-specific analyses. This limitation is relevant in the Japanese context, where traditional martial arts and Western-influenced sports might show distinct leadership dynamics and developmental patterns.

Furthermore, since both the YES-S and DTLI-YS measures were originally developed outside of Asian contexts, their measurement properties deserve careful examination when applied in different cultural settings, especially regarding individual vs. collectivist orientations. The potential heterogeneity in participants’ cultural backgrounds, despite the predominantly Japanese sample, may have influenced the results and merits additional investigation.

Future research should address these limitations by developing methods to directly measure cultural factors, conducting longitudinal studies to establish causality, and validating findings across diverse age groups and competitive levels.

## 5 Conclusion

This study reveals relationships between TFL and youth development outcomes that appear distinctive within Japanese cultural contexts. High performance expectations showed significant relationships with both goal setting (*β* = .29, *p* < .05) and initiative (*β* = .39, *p* < .01), reflecting potential distinctive characteristics of Japanese sports coaching environments. Additionally, our study found a moderate positive correlation between overall TFL behaviors and PYD outcomes (*r* = .49, *p* < .01), supporting the general association between transformational approaches and positive development in Japanese contexts. This pattern is notable when compared to American research where correlations between PYD outcomes and TFL dimensions were typically weaker (*r* = .11–.43), suggesting the potential role of Japan's collectivist values and hierarchical relationships on leadership effectiveness.

Our findings also revealed a noteworthy contrast in leadership effectiveness patterns, with Japanese athletes responding more strongly to coaches’ high expectations than to their role modeling behaviors. This suggests cultural differences in how leadership influences operate within coach-athlete relationships, with hierarchical expectations potentially functioning effectively even without strong personal identification with coaches as role models.

Analysis by competition level revealed significant differences in initiative (*F* = 4.07, *p* < .01, *η*^2^ = .10) and YES-S total scores (*F* = 2.75, *p* < .05, *η*^2^ = .07), suggesting associations between competitive advancement and developmental outcomes. These findings indicate that success at higher competitive levels involves both appropriate expectation-setting by coaches and athletes’ autonomous engagement.

These findings extend previous research on youth sports coaching (33, 34) by demonstrating the effectiveness of expectation-centered approaches within Japanese cultural contexts. However, the results also indicate the importance of developing comprehensive approaches that incorporate leadership elements beyond expectation-setting.

## Data Availability

The raw data supporting the conclusions of this article will be made available by the authors, without undue reservation.
